# Supplementary material for: Developing and Applying Heterogeneous Phylogenetic Models with XRate
Source: PLoS One. 2012 Jun 5;7(6):e36898. doi: 10.1371/journal.pone.0036898 (PMC3367922; doi:10.1371/journal.pone.0036898)
Supplement: Text S2 — contains tables describing the Scheme- XRate functions available in Darts. (PDF) [file pone.0036898.s002.pdf]

Tables of Scheme functions in Darts

The following list of Scheme functions, natively implemented within selected DART programs (including XRate) when compiled with GNU Guile, is only complete to the date of publication. A more up-to-date list may be found at `http://biowiki.org/DartSchemeFunctions`

Darts functions for working with trees

Scheme function	Effect
(newick-from-string x)	Create a tree-smob from a Newick-format string x
(newick-from-file x)	Create a tree-smob from a file x
(newick-from-stockholm x)	Create a tree-smob from the tree encoded within alignment-smob x
(newick-to-file x y)	Write tree-smob x to file y in Newick format
(newick-ancestor-list x)	List of all ancestors in the tree-smob x
(newick-leaf-list x)	List of all leaves in the tree-smob x
(newick-branch-list x)	List of all branches in the tree-smob x
(newick-unpack x)	Converts a tree-smob x into a Scheme data structure

Darts functions for working with alignments

Scheme function	Effect
(stockholm-from-string x)	Create an alignment-smob from a Stockholm-format string x
(stockholm-from-file x)	Create an alignment-smob from a Stockholm-format file x
(stockholm-to-file x y)	Write alignment-smob x to Stockholm-format alignment file y
(stockholm-column-count x)	Return the number of columns in alignment-smob x
(stockholm-unpack x)	Converts an alignment-smob x into a Scheme data structure

Functions for working with grammars

Scheme function	Effect
(xrate-validate-grammar x)	Validate the syntax of XRate grammar x
(xrate-validate-grammar-with-alignment x y)	Validate the syntax of XRate grammar x, using alignment-smob y to expand macro constructs
(xrate-estimate-tree x y)	Use XRate grammar y to estimate a tree for alignment-smob x
(xrate-annotate-alignment x y)	Use XRate grammar y to annotate alignments-smob x
(xrate-train-grammar x y)	Train XRate grammar y on the list of alignment-smobs y

Miscellaneous functions

Scheme function	Effect
(dart-log x)	Logging directive; equivalent to "`-log x`" at the command line
(discrete-gamma-medians alpha beta K)	Returns the median rates of K equal-probability bins of the gamma distribution
(discrete-gamma-means alpha beta K)	Returns the mean rates of K equal-probability bins of the gamma distribution
(ln-gamma k)	Calculates the gamma function, $\Gamma(k) = \int_0^\infty e^{-x} x^{k-1} dx$
(gamma-density x alpha beta)	Calculates the gamma probability density, $\beta^\alpha \frac{1}{\Gamma(\alpha)} x^{\alpha-1} e^{-\beta x}$
(incomplete-gamma x alpha beta)	Calculates the incomplete gamma function, i.e. the integral of the gamma density up to x
(incomplete-gamma-inverse p alpha beta)	Calculates the inverse of the incomplete gamma function
