# Supplementary material for: Developing and Applying Heterogeneous Phylogenetic Models with XRate
Source: PLoS One. 2012 Jun 5;7(6):e36898. doi: 10.1371/journal.pone.0036898 (PMC3367922; doi:10.1371/journal.pone.0036898)
Supplement: Text S3 — contains a glossary of XRate terminology. (PDF) [file pone.0036898.s003.pdf]

Glossary of XRate terminology

Within the glossary descriptions, *italicized phrases* refer to other glossary terms.

Alignment: See *multiple sequence alignment.*

Alphabet: The set of single-character tokens (symbols) from which sequences are constituted. The alphabet is defined in the grammar file; only one alphabet may be defined per grammar file. (Usually the alphabet is DNA, RNA or protein. Sometimes the alphabet is extended to include an explicit gap character.) An alphabet may optionally include a *complement* mapping, as well as specification of degenerate (ambiguous) characters.

Ancestral reconstruction: The use of XRate to reconstruct the sequences at ancestral nodes of a *phylogenetic tree*, given a *grammar*, a *multiple sequence alignment* and a *parse tree.* This occurs after *tree estimation*, *training* and *annotation.*

Annotation: The use of XRate to apply a *grammar* to a *multiple sequence alignment* and *phylogenetic tree*, so as to impute the optimal *parse tree* and mark up the alignment with the co-ordinates of selected features (associated with particular *nonterminals* in the parse tree), or generate other annotation including GFF and WIGgle files. This occurs after *tree estimation* and *training*, but prior to *ancestral reconstruction.* Can also refer to a specific part of a *transformation rule* that generates annotations.

Bifurcation: A *transformation rule* that generates two *nonterminals.* Bifurcation rules have the form

```
(transform (from (A)) (to (B C)))
```

where `A`, `B` and `C` are nonterminals.

Chain: A substitution rate matrix (the name comes from "continuous-time Markov chain"). The states of the substitution process are N-mers augmented with an optional hidden variable. That is, the state space of a chain consists of *state-tuples* of the form $(s_1, s_2, \ldots, s_N, h)$ with $N \geq 1$, where s_1 through s_N represent *alphabet* symbols (which will be observed in the final *multiple sequence alignment*) and h is an optional *hidden state* which can take on a finite set of single-character values specific to this chain. Each of the N alphabet symbols, s_1 through s_N, is associated with a unique *pseudoterminal.* Examples of valid chain state spaces include the set of all nucleotides; the set of all codons; and the set of all tuples (A, H) where A is an amino acid and $H \in \{F, S\}$ is a hidden binary variable taking values F (for fast) or S (for slow).

Complement: An order-2 permutation on the *tokens* of an *alphabet.* (Typically only used for DNA or RNA alphabets.)

Emission: A *transformation rule* that generates some *pseudoterminals* (and thus, some alignment columns); or the set of pseudoterminals (or alignment columns) generated by such a rule. In XRate, emission rules have

the form $A \to x_1 \ldots x_L\ A*\ x_{L+1} \ldots x_{L+R}$ where $A, A*$ are paired *nonterminals* (whose names differ only by the final asterisk) and $x_1 \ldots x_{L+R}$ are pseudoterminals. ($A*$ is referred to as the *post-emit nonterminal.*) Any numbers L, R of pseudoterminals can appear to the left and right of the $A*$, as long as $L + R > 0$. If $L = 0$ and $R > 0$, the rule is a *right-emission*; if $L > 0$ and $R = 0$, the rule is a *left-emission.* The pseudoterminals $x_1 \ldots x_{L+R}$ must comprise (any permutation of) the full set of pseudoterminals for a given *substitution chain.* Each pseudoterminal may optionally be prefixed with a tilde character (~) to indicate that it should be *complemented* in the final alignment (used to generate reverse strands in double-stranded models). For example, if `C1`, `C2` and `C3` are the three pseudoterminals of a codon chain, `A` is an emission nonterminal and `A*` is the corresponding post-emission nonterminal, valid emission rules could include

```
(transform (from (A)) (to (C1 C2 C3 A*)) (prob (...)))
```

and

```
(transform (from (A)) (to (~C3 A* ~C2 ~C1)) (prob (...)))
```

Grammar: The contents of a grammar file: *chains*, *nonterminals*, *transformation rules*, and *alphabet.* (The alphabet is specified in a separate part of the file from the rest of the grammar, and so is sometimes omitted from this definition.)

Grammar symbol: A symbol that is either a *nonterminal* or a *pseudoterminal.*

HMM: Hidden Markov Model. An *SCFG* that is also a *regular grammar.* See also *phylo-HMM.*

Hidden state: In the context of XRate, this term is ambiguous (see *state*). In this article, it is used mostly to refer to the final element of a *state-tuple* in a *chain.* However, in the context of *HMM* theory, it refers to what we call a *nonterminal.*

Hybrid chain: A mapping from tree branches to substitution rate matrices (*chains*) where the instantaneous rate matrix may vary from one branch to another. This may be used to implement lineage-dependent selection, or other models which are heterogeneous with respect to the tree.

Initial distribution: The initial probability distribution over states in a substitution *chain.*

Left-emission: See *emission.*

Left-regular: A *grammar* is left-regular if it contains no *bifurcations* and its *emissions* are all *left-emissions.*

Macro: A construct that is expanded by the XRate grammar preprocessor and may be used to implement redundant or repetitive grammar models; e.g.

grammars with a large number of similar *transformation rules* sharing the same probability parameter, or substitution *chains* whose *mutation rules* all share the same rate parameter.

Multiple sequence alignment: The raw data on which XRate operates, and which constitutes its input and output. XRate cannot align sequences, but assumes that they have been pre-aligned using an external alignment program. Alignments must be converted to Stockholm format [**?**] before supplying them to XRate. The alignment may include a *phylogenetic tree* (using the Stockholm syntax for specifying this); if no tree is provided, XRate's *tree estimation* routines can be used to find one.

Mutation rule: A single element in the rate matrix of a substitution *chain*.

Nonterminal: A *grammar symbol* that may be transformed, by application of *transformation rules*, into other nonterminals or pseudoterminals. In XRate, a nonterminal must be exclusively associated with (that is, appear on the left-hand side of) either *emission* rules, *transition* rules or *bifurcation* rules.

Parameter: A named parameter in a grammar. May be a *probability parameter* or a *rate parameter*.

Parametric model: A *grammar* whose *transformation rules* or *mutation rules* (or both) are specified as functions of the grammar's *parameters*, rather than as direct numerical values.

Parse tree: A tree structure corresponding to the derivation of a multiple sequence alignment from a *grammar*. Each tree node is labeled with a *grammar symbol*: the root node is labeled with the *start nonterminal*, internal nodes are labeled with *nonterminals*, and the leaves are labeled with *pseudoterminals*. Not to be confused with a *phylogenetic tree*.

PGroup: A set of *probability parameters* collectively representing a probability distribution over a finite set of events. Following training, probability parameters constituting a PGroup will be normalized to sum to 1.

Phylogenetic tree: The evolutionary tree describing the relationship between sequences in a multiple alignment. XRate uses the Stockholm format for alignments, which allows the tree to be included as an annotation of the alignment. If no tree is provided, XRate's *tree estimation* routines can be used to find one.

Phylo-grammar: See *phylo-SCFG*.

Phylo-HMM: A *phylo-SCFG* that uses a *regular grammar*. A phylo-HMM is an HMM whose *emissions* generate alignment columns by evolving *substitution chains* on a phylogenetic tree.

Phylo-SCFG: A phylogenetic *SCFG*: a member of the general class of *grammars* implemented by XRate. A phylo-SCFG is an SCFG whose *emissions* generate alignment columns by evolving *substitution chains* on a phylogenetic tree.

Post-emit nonterminal: See *emission.*

Production rule: See *transformation rule.*

Probability parameter: A dimensionless *parameter* that generally takes a value between 0 and 1, and so can occur in the probability part of a *transformation rule* (or as a multiplying factor in the rate part of a *mutation rule*). Probability parameters are declared in *PGroups.*

Pseudocounts: A set of nonnegative counts that specifies a Dirichlet prior distribution over a *PGroup.*

Pseudoterminal: A *grammar symbol* that is generated via an *emission* and cannot be further modified by subsequent *transformation rules.* In a *parse tree*, a pseudoterminal serves as a placeholder for an alignment column. Pseudoterminals occur in groups associated with a particular *substitution chain.* In the generative interpretation of the model, alignment columns are generated using the *initial distribution* and *mutation rules* of the chain, applied on the phylogenetic tree associated with the alignment.

Rate parameter: A nonnegative parameter that has units of "inverse time" (i.e. rate), and so can occur in the rate part of a *mutation rule.* Rate parameters can be declared individually.

Regular grammar: A *grammar* is regular if it is either *left-regular* or *right-regular*; that is, it contains no *bifurcations* and its *emissions* are all either *left-emissions* or *right-emissions.* A regular grammar is equivalent to an *HMM.*

Right-emission: See *emission.*

Right-regular: A *grammar* is right-regular if it contains no *bifurcations* and its *emissions* are all *right-emissions.*

SCFG: Stochastic Context-Free Grammar. See also *phylo-SCFG.*

Start nonterminal: The first *nonterminal* declared or used in a grammar. In the generative interpretation of the model, this is the initial *grammar symbol* to which transformation rules are applied. It is also the label of the root node in the *parse tree.*

State: In the context of a *phylo-grammar*, this term is ambiguous: it can refer either to a *state-tuple* in a *chain*, or (for phylo-HMMs) a *nonterminal* in a *grammar.* For the most part in this paper, and exclusively in this glossary, we use it in the former sense.

State space: The set of possible *state-tuples* in a *chain.*

State-tuple: A tuple of the form $(s_1, s_2, \ldots, s_N, h)$ representing a single state in a *chain*, where s_1 through s_N represent *alphabet* symbols and h is an optional *hidden state.*

Substitution chain: A continuous-time finite-state Markov chain over *state-tuples.* See *chain.*

Substitution model: See *substitution chain.*

Terminal: See *token.*

Token: An *alphabet* symbol. (Also called a *terminal.*)

Training: The use of XRate to estimate a grammar's *parameters*, *mutation rule* rates and *transformation rule* probabilities, given a (set of) multiple alignments. This occurs after *tree estimation* and prior to *annotation* or *ancestral reconstruction.*

Transformation rule: A probabilistic rule that describes the transformation of a *nonterminal* symbol into a sequence of zero or more *grammar symbols.* (Also called a *production rule.*) A transformation rule may be an *emission*, a *transition* or a *bifurcation.*

Transition: A *transformation rule* that generates exactly one nonterminal (and no pseudoterminals). Transition rules have the form

```
(transform (from (A)) (to (B)) (prob (...)))
```

where `A` and `B` are nonterminals.

Tree: In the context of a *phylo-grammar*, this term is ambiguous: it can mean a *parse tree* (which explains the "horizontal", i.e. spatial, structure of an alignment) or a *phylogenetic tree* (which explains the "vertical", i.e. temporal, structure).

Tree estimation: The use of XRate to estimate a *phylogenetic tree* for a *multiple sequence alignment*, given a *grammar.* This occurs prior to *training*, *annotation* or *ancestral reconstruction.*
